# Relationships between Cell Cycle Regulator Gene Copy Numbers and Protein Expression Levels in *Schizosaccharomyces pombe*

**DOI:** 10.1371/journal.pone.0073319

**Published:** 2013-09-03

**Authors:** Ayako Chino, Koji Makanae, Hisao Moriya

**Affiliations:** 1 Graduate School of Science and Technology, Okayama University, Kita-ku, Okayama, Japan; 2 Research Core for Interdisciplinary Sciences, Okayama University, Kita-ku, Okayama, Japan; University College London, United Kingdom

## Abstract

We previously determined the copy number limits of overexpression for cell division cycle (*cdc*) regulatory genes in the fission yeast *Schizosaccharomyces pombe* using the “genetic tug-of-war” (gTOW) method. In this study, we measured the levels of tandem affinity purification (TAP)-tagged target proteins when their copy numbers are increased in gTOW. Twenty analyzed genes showed roughly linear correlations between increased protein levels and gene copy numbers, which suggested a general lack of compensation for gene dosage in *S. pombe*. Cdc16 and Sid2 protein levels but not their mRNA levels were much lower than that expected by their copy numbers, which suggested the existence of a post-transcriptional down regulation of these genes. The cyclin Cig1 protein level and its mRNA level were much higher than that expected by its copy numbers, which suggested a positive feedback mechanism for its expression. A higher Cdc10 protein level and its mRNA level, probably due to cloning its gene into a plasmid, indicated that Cdc10 regulation was more robust than that previously predicted.

## Introduction

We previously developed a genetic method called “genetic Tug-Of-War (gTOW)” to determine the copy number limit of overexpression of target genes in budding and fission yeasts [1]–[3]. In gTOW, a target gene with its native promoter and terminator is cloned into a multicopy plasmid, and the plasmid copy number is increased by up to 100 copies per cell using a selection bias arising from a truncated *leu2* gene on the plasmid. If the target gene has a copy number limit for overexpression, the copy number of this plasmid must be less than this limit. The resulting tug-of-war between the bias to increase the copy number arising from the *leu2* gene and to decrease the copy number arising from the target gene determines the plasmid copy number in a cell. This number should be close to the upper limit for the target gene. Using gTOW, we previously determined the copy number limits for 32 cell division cycle (cdc) regulatory genes in the fission yeast *Schizosaccharomyces pombe* [2]. These limits ranged from <2 copies to >100 copies per haploid genome. Using these data, we refined an integrative mathematical model for the fission yeast cell cycle [2].

A major drawback of gTOW is that the level of protein expressed from the target gene may not be linearly correlated with its copy number. However, the existence of some regulatory mechanisms within the gene/protein expression system is evident if there is an inconsistency between the copy number and protein level. If there is a strong negative feedback within the expression system of a protein, the protein level will not increase with an increase in copy number [4], [5], whereas if there is a positive feedback, the protein level will dramatically increase with an increase in copy number. Some other mechanisms, such as RNA interference (RNAi), may also influence the correlations between protein levels and gene copy numbers [6].

Thus, in this study, we measured the protein levels with an increase in copy number by gTOW to determine the correlations between protein levels and gene copy numbers in *S. pombe*.

## Results

### Construction of Plasmids and Yeast Strains Used for Measuring Protein Levels

**Figure 1:**
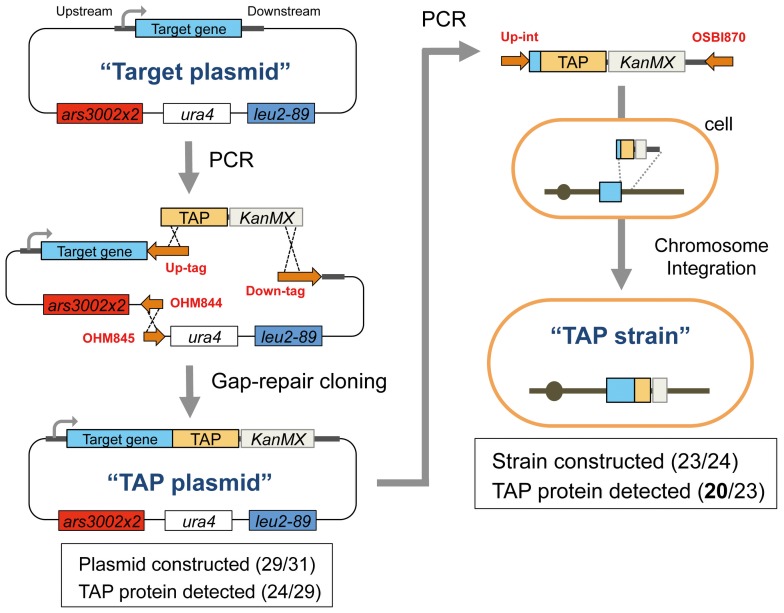
Procedures for TAP plasmid and TAP strain construction. We first constructed a “TAP plasmid” from a “Target plasmid” containing one of the target cdc genes [2]. This TAP plasmid was then used as a PCR template for the chromosomal integration of the TAP construct to obtain the “TAP strain.” Red letters indicate the PCR primers, which are listed in Tables S6, S7, S8, and S9. The details for construction are described in the Strains and plasmids section of Methods. The numbers of successfully constructed plasmids and TAP protein detection are also shown.

As targets for this study, we chose 31 *cdc* genes that we had previously analyzed by gTOW [2]. These are listed in Table S1. Because specific antibodies against most of these Cdc proteins were not available, we used tandem affinity purification (TAP) tagging to detect the target proteins of interest [7]. We first attempted to construct C-terminally TAP-tagged *cdc* genes on a gTOW plasmid (plasmid construction is shown in Figure 1). We had gTOW vectors with three different maximum plasmid copy numbers; we chose the “middle” vector because it covered the widest range for copy number limits [1]–[3]. Each tagged-target protein was expressed using its native promoter, although each terminator was not native, as we used the terminator for the *ADH1* gene from *S. cerevisiae*. We designated the constructed plasmid TAP plasmid. Of 31 genes that we attempted to analyze, we successfully constructed 29 TAP plasmids and confirmed the expressions of 24 TAP proteins using these plasmids (summarized in Figure 1 and Table S1).

**Figure 2:**
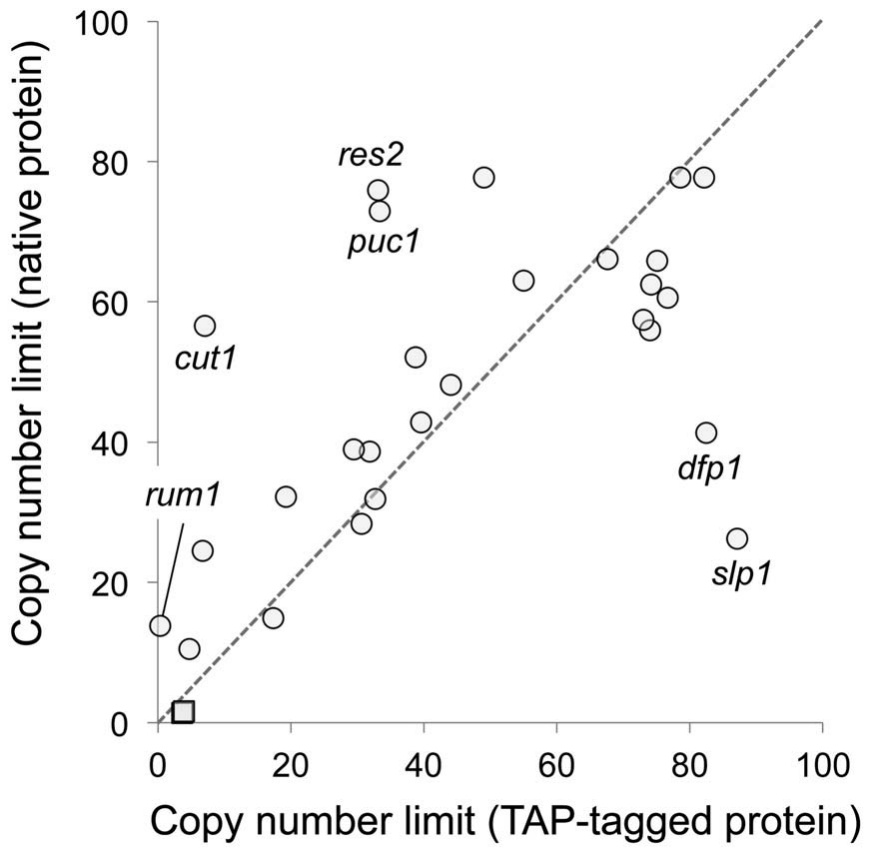
Relationships between native and TAP-tagged gene copy number limit. Genes whose copy numbers varied between native and TAP-tagged are shown. Circles indicate those genes whose copy numbers were determined under −leucine conditions, and squares indicate genes whose copy numbers were determined under +leucine conditions. Copy numbers of native genes were obtained from previously published results [2]. The averages of more than three independent experiments are shown. The original data with standard deviations are provided in Table S2.

Because C-terminal TAP-tagging may affect the activity of target proteins, we indirectly evaluated their activities by measuring the copy number limits of tagged genes and compared these with those of native genes. As shown in Figure 2, the tagged genes and the native genes exhibited similar overall copy number limits (Pearson’s *r* = 0.65). However, the tagged genes *dfp1* and *slp1* showed increased copy number limits compared with their native genes, which suggested that their activities were reduced. In contrast, the tagged genes *cut1*, *puc1*, *res2*, and *rum1* showed reduced copy number limits compared with their native genes, which suggested that their activities were increased or that they had become dominant active.

We further replaced each target gene on the *S. pombe* chromosome with the same TAP-tagged gene in a TAP plasmid so that we could detect the target protein expressed by one copy of the target gene (strain construction is shown in Figure 1). We designated this yeast strain as TAP strain. Of 24 genes, we successfully constructed 23 TAP strains and confirmed their expressions of 20 TAP proteins (summarized in Figure 1 and Table S1).

### Quantifying Proteins Expressed by High-copy Plasmids

**Figure 3:**
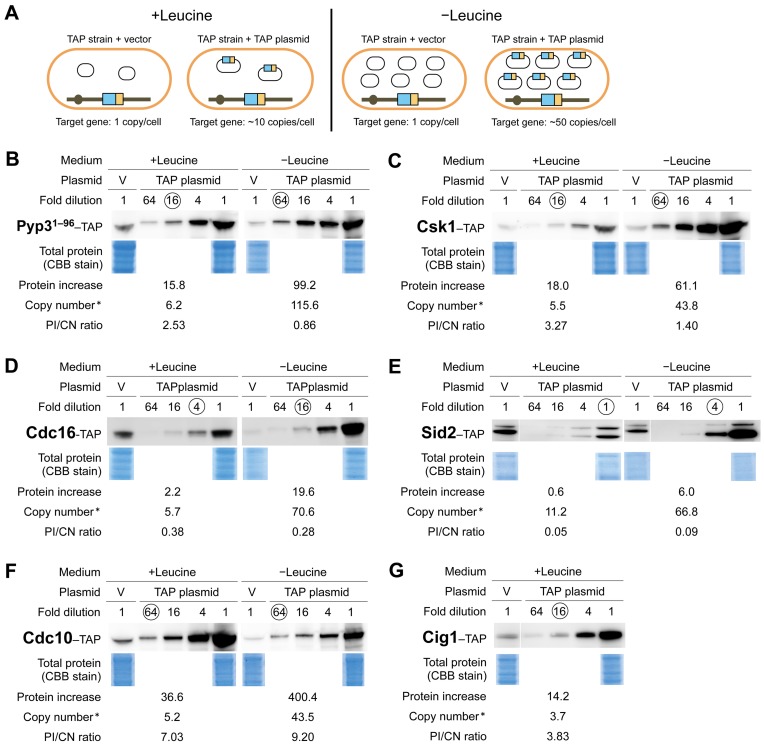
Quantifying Cdc–TAP protein levels with increased gene copy numbers. **A**. *S. pombe* strains for determining increased protein levels expressed by a TAP plasmid and a chromosomal copy with an increase in gene copy number. Each *cdc–*TAP strain was transformed with either an empty vector or the corresponding *cdc–*TAP plasmid and then cultured in medium with or without leucine. **B**–**E** are examples of these quantitative results. The levels of TAP-tagged proteins were determined as described in Methods. Copy number* indicates the copy number of each TAP plasmid plus 1 (chromosomal copy). Circled numbers indicate the fold-dilutions used to measure the intensity of a Cdc–TAP protein. Total proteins were visualized using Coomassie® G-250 staining. **B**. Pyp3^1–96^–TAP used as a control. **C**. Csk1–TAP; an example for which the protein level increase and the copy number were well correlated. **D** and **E**. Cdc16–TAP and Sid2–TAP; examples for which the protein levels did not increase with an increase in copy number. **F** and **G**. Cdc10–TAP and Cig1–TAP; examples for which protein level increases exceeded copy number increases. All Cdc–TAP analyses are shown in Figure S2 and the quantitative results are summarized in Table 1.

We then measured each of the TAP-tagged Cdc proteins expressed in the TAP strains harboring an empty vector or a TAP plasmid cultured in medium with or without leucine using quantitative Western blotting (Figure 3A; procedure shown in Figure S1). Our aim was not to quantify the absolute levels of these target proteins but to measure the fold increase in the protein level when expressed by the TAP plasmids over that expressed by the genomic copy (single copy). To avoid saturation effects with quantitative Western blotting, we prepared serial dilutions of the proteins expressed in the strains with TAP plasmids. Examples of these measurements are shown in Figure 3 and all measurements are shown in Figure S2.

To assess the reproducibility of these measurements, we performed control experiments using a truncated pyp3–TAP construct (Pyp3^1–96^–TAP), which we have accidentally constructed. We fused a TAP tag immediately after the 1^st^ exon of Pyp3 to create a fusion protein containing a part of Pyp3 (96 amino acids) of its total of 303 amino acids. Because most of the protein phosphatase domain of Pyp3 was removed by this procedure, we considered that this TAP-tagged protein has no activity. The results of one of the four independent experiments with Pyp3^1–96^–TAP are shown in Figure 3B.

**Figure 4:**
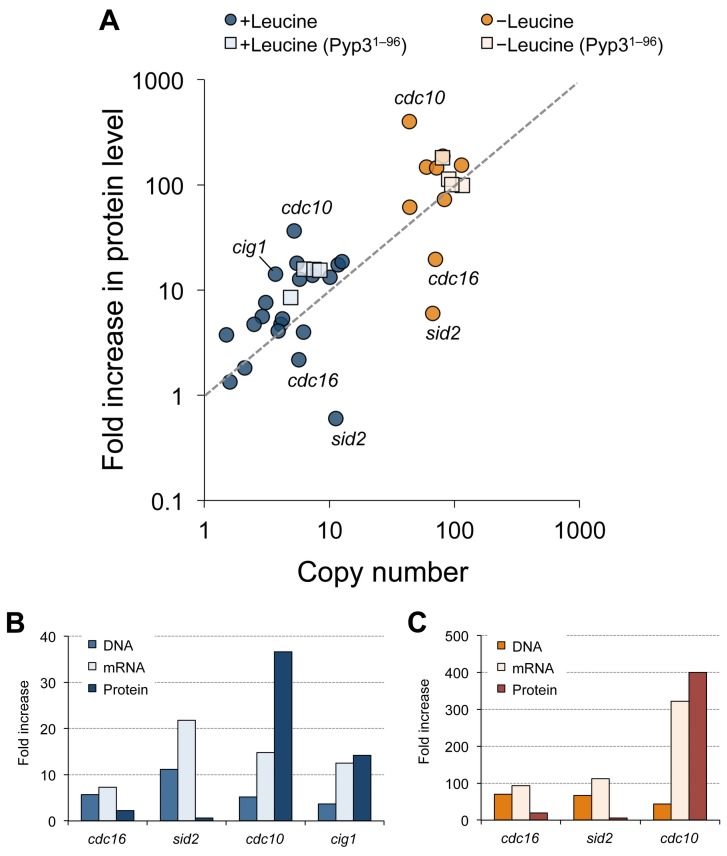
Relationships between fold-increases in protein levels and copy numbers. **A**. A scatter plot between the fold increase in protein level and the copy number. Squares indicate the results of control experiments using Pyp3^1–96^–TAP. Genes that showed high variations between protein level increases and copy numbers are indicated. **B** and **C**. Fold increase in the gene copy number, the mRNA and protein levels of indicated gene in the +leucine (**B**) and –leucine (**C**) conditions. The original data for the gene copy number and protein increase are provided in Table 1, and the data of the mRNA increase are provided in Table S3.

We simultaneously measured the copy number (CN) of TAP plasmid in a cell for each experiment and compared this with the fold-increase in the protein level (protein increase: PI) measured as described above. We then calculated the PI/CN ratio. This corresponded to the protein level expressed by each copy of the gene when the gene copy number increased (Table 1). Figure 4A shows the relationships between copy numbers and protein increases. As expected, the protein levels increased according to the increase in copy number in four control experiments with Pyp3^1–96^–TAP. We did not analyze the protein levels of genes those have the copy number limits less than the copy number of the middle vector (about 55 copies/cell) in −leucine conditions [2], because the cells with the TAP plasmids of these genes did not grow well in −leucine conditions and we thus had difficulties to prepare equal amount of cells and extracted proteins to the control ones for the quantifications of TAP-tagged proteins (data not shown).

**Table 1:** Relationships between copy numbers and fold increase in protein level.

	+Leucine			−Leucine		
	Protein increase	Copy number*	PI/CN Ratio	Protein increase	Copy number*	PI/CN Ratio
*ark1*	4.0	6.2	0.64	147.9	59.5	2.49
*cdc7*	1.3	1.6	0.83	ND	ND	ND
*cdc10*	36.6	5.2	7.03	400.4	43.5	9.20
*cdc16*	2.2	5.7	0.38	19.6	70.6	0.28
*cdc18*	7.6	3.1	2.47	ND	ND	ND
*cdc25*	5.6	2.9	1.93	ND	ND	ND
*chk1*	13.2	10.1	1.31	187.2	80.9	2.31
*cig1*	14.2	3.7	3.84	ND	ND	ND
*cig2*	4.7	2.5	1.89	ND	ND	ND
*clp1*	1.8	2.1	0.87	ND	ND	ND
*csk1*	18.0	5.5	3.27	61.1	43.8	1.40
*cut2*	4.7	4.1	1.16	ND	ND	ND
*fkh2*	5.3	4.2	1.26	ND	ND	ND
*hsk1*	17.3	11.7	1.48	153.8	114.0	1.35
*mik1*	13.8	7.3	1.89	ND	ND	ND
*plo1*	4.1	3.9	1.04	ND	ND	ND
*ras1*	18.6	12.6	1.47	72.7	83.1	0.87
*rum1*	3.7	1.5	2.49	ND	ND	ND
*sid2*	0.6	11.2	0.05	6.0	66.8	0.09
*pyp3*	12.7	5.8	2.20	145.1	72.0	2.02
*pyp3^1–96^*-1	15.8	6.2	2.53	99.2	115.6	0.86
*pyp3^1–96^*-2	15.7	7.5	2.08	180.2	79.8	2.26
*pyp3^1–96^*-3	8.5	4.9	1.74	113.1	89.6	1.26
*pyp3^1–96^*-4	15.4	8.4	1.83	100.2	95.4	1.05

ND: Not done,

* The number shown is the plasmid copy number determined plus 1 (genomic copy).

Overall, 20 Cdc–TAP proteins showed some correlation between copy numbers and fold increases in protein levels (Pearson’s *r* = 0.57), although variations were observed, particularly with +leucine conditions (Figure 4A). We found no general trend, such as compensation for the gene dosage. Among the *cdc* genes analyzed, *cdc16* and *sid2* had low PI/CN ratios (Figures 3D, 3E, and 4A), whereas *cdc10* and *cig1* had high PI/CN ratios (Figures 3F, 3G, and 4A).

### Quantifying mRNA Expressed by High-copy Plasmids

We next measured the mRNA levels of the 4 TAP-tagged gene mentioned above using real-time PCR to examine if the low or high PI/CN ratios arose from their mRNA levels (Figure S1). We calculated fold increase in the mRNA level for each target gene expressed in the cells of the TAP strain harboring the TAP plasmid over the cells of TAP strain harboring the empty vector. The results of measurements are shown in Table S3. Figure 4B and 4C indicate the gene copy numbers (DNA), and the fold increases in the mRNA and protein levels. In the cases of *cdc16* and *sid2*, mRNA levels increased when their gene copy numbers increased, but protein levels did not increase in the same situation. In the cases of *cdc10* and *cig1*, mRNA levels dramatically increased when their gene copy number increased, and the levels were similar to the levels of their proteins.

### Quantifying Proteins Expressed by the Chromosomal Genes when their Gene Copy Numbers Increase

**Figure 5:**
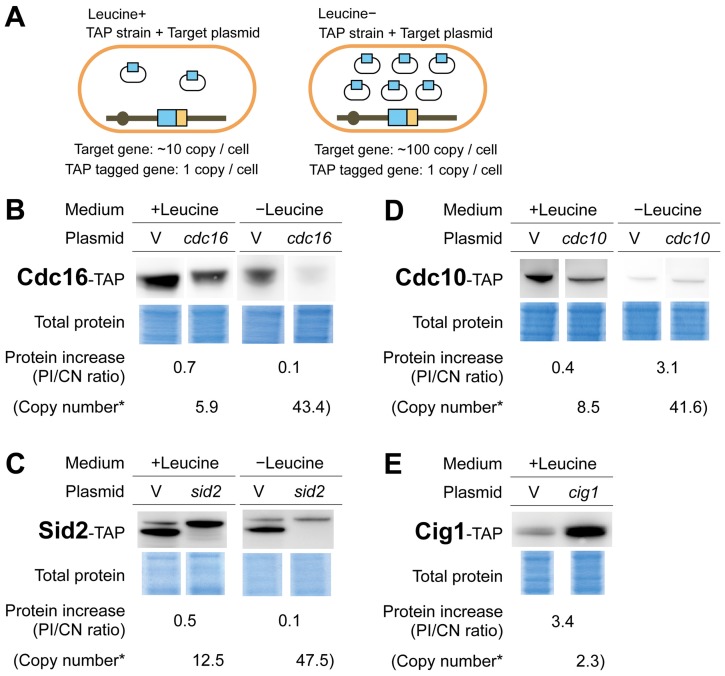
Quantifying Cdc–TAP protein levels expressed by a single chromosomal copy with an increase in gene copy number. **A**. *S. pombe* strains for determining the increases in protein levels expressed by a single chromosomal copy with an increase in gene copy number. Each *cdc–*TAP strain was transformed with either an empty vector or the corresponding target plasmid and then cultured in medium with or without leucine (as indicated). **B**–**E**. Quantitative results for Cdc16–TAP, Sid2–TAP, Cdc10–TAP, and Cig1–TAP. TAP-tagged protein levels were determined as described in Methods. Copy number* is the copy number of a Target plasmid plus 1 (chromosomal copy).

We further measured the levels of the 4 proteins mentioned above when they were expressed by the chromosomal genes to confirm if the expression by each gene copy also changed when the gene copy numbers increased. As shown in Figure 5A, we measured the TAP-tagged Cdc proteins expressed by the chromosomal genes of the TAP strains when the target plasmids (without TAP-tag) were introduced. We then compared these protein levels with those expressed by the chromosomal genes of the TAP strain with the empty vector. As shown in Figures 5B and 5C, Cdc16–TAP and Sid2–TAP expressed by the chromosomal genes were dramatically reduced when their gene copy numbers increased, suggesting that the levels of these proteins were compensated by increase in the numbers of their genes. Cig1–TAP protein expressed by the chromosomal gene increased when its copy number increased (Figure 5E), suggesting the existence of positive feedback mechanism for Cig1 protein expression. However, Cdc10–TAP expressed by a chromosomal gene did not show consistent higher levels when the gene copy number increased (Figure 5D), and the PI/CN ratios (0.4 and 3.1) were much lower than the ones expressed from the TAP plasmid (7.03 and 9.20, Figure 3F). Thus, we considered that the high PI/CN ratio observed with Cdc10–TAP plasmid experiment (Figure 3F) was not because of a feedback mechanism but because of an artificial increase in expression after cloning *cdc10* on a plasmid.

## Discussion

For this study, we used the gTOW method and quantitative Western blotting with TAP-tagged proteins to measure protein levels when the copy numbers of the respective genes were increased in *S. pombe*. The protein levels and copy numbers were roughly linearly correlated (Figure 4A). We previously obtained similar results for budding yeast [1]. In budding yeast, a general lack of compensation for gene dosage was suggested by protein measurements for heterozygous deletion mutants and aneuploids [8]–[10]. Together, our results indicate that there is no general buffering/compensatory mechanism in response to a change in gene copy number in yeasts.

However, we did find several genes that exhibited mismatches between their copy numbers and the fold increases in their proteins. These results suggested gene/protein specific compensation for gene dosage or feedback mechanisms. Both Cdc16 and Sid2 showed decreases in the protein levels expressed by their single chromosomal genes with increased gene copy numbers with increased gene copy numbers (Figures 5B and 5C). Because mRNA levels of these genes were increased when their genes copy numbers increased (Figures 4B and 4C), the lower protein levels should be explained by their post-transcriptional regulations. Together with Byr4, Cdc16 constitutes a bipartite G-protein activating enzyme, G-protein Spg1 [11]. Byr4 degradation is accelerated when its binding to Spg1 is disrupted [12]. Sid2 is a protein kinase, which constitutes an active complex with Mob1 [13]. All of these proteins are involved in a signaling network termed the “septation initiation network” and constitute a large protein complex on the spindle pole body [14]. We suggest that Cdc16 and Sid2 degradation may be accelerated when there is a stoichiometric imbalance with their partners, as there is with Byr4. Cdc16 and Sid2 have relatively high copy number limits (>100 copies) [2]. The strong compensation for gene dosage found in this study could be one of the reasons for these high limits.

The increased expression of Cig1 by a single chromosomal gene with an increase in gene copy number suggests a positive feedback mechanism for the Cig1 expression system. Because the increase in the mRNA level was also higher than that expected by its copy number increase, the positive feedback mechanism would be functioning in the transcript level. Currently, no positive feedback regulation in the transcription of *cig1* has reported. Further investigation is required to uncover the regulation.

Cdc10–TAP expressed by its plasmid had a higher PI/CN ratio (7.07 and 9.20 under +leucine and –leucine conditions, respectively, Figure 3F); however, that expressed by its genome did not have a high PI/CN ratio (below 3.1 fold, Figure 5D). This indicated that *cdc10* expression regulation by its plasmid was different from that by its genome. The off-state transcription of a uracil-regulatable gene *urg1* is significantly increased after plasmid cloning [15]. *cdc10* may be regulated in a similar locus-specific manner.

We previously refined a mathematical model for the fission yeast cell cycle based on copy number limits determined by gTOW [2]. We increased the limit for *cdc10* from the original model so that the model could reproduce the robustness of *cdc10* regulation. However, in this study, we found that the Cdc10 protein limit was much higher than that expected from the gene copy number (Figure 3F). The fission yeast cell cycle may be insensitive to strong Cdc10 overexpression because the function of Cdc10 as a transcription factor is in conjunction with other factors, such as Res1, Res2, Nrm1, and Yox1 [16]–[18]. These were not incorporated in our current mathematical model.

## Methods

### Growth Media, Culture Conditions, and DNA Preparations

*S. pombe* cell cultivation, yeast transformation, yeast DNA isolation (yeast DNA miniprep), and DNA minipreps from *E. coli* were performed as described previously [19].

### Strains and Plasmids

The plasmids and yeast strains used in this study are listed in Table S4 and Table S5, respectively. TAP plasmids and TAP strains were constructed as shown in Figure 1. All primers used to construct plasmids and strains are listed in Tables S6, S7, S8, and S9. For each construct, two DNA fragments were amplified by polymerase chain reaction (PCR) with Up-tag and Down-tag primers using each pTOWsp-M-target plasmid [2] as a template. A TAP tag–*KanMX* cassette, which was constructed from pKT232 [20] and pTOWug2-ESP1-TAP [21], was amplified by PCR with the primers OHM179 and OHM182. All three DNA fragments were combined by homologous recombination activity in *S. pombe* as described previously [19]. This procedure also removed the unnecessary *his5MX* gene from the vector, which resulted in a vector backbone identical to pTOWspd5-M [3]. Each constructed plasmid was recovered from *S. pombe* cells, and its structure was checked by restriction enzyme digestion and partial sequencing. To construct TAP strains, a TAP–*KanMX* cassette containing parts of the coding region for a target gene and the 3′ region for homologous recombination was amplified by PCR with Up-int and OSBI870 primers using each TAP plasmid as a template and then introduced into *S. pombe* cells to integrate the cassette into the genomic region of the target gene. G418 (final conc.: 150 mg/mL) was used for *KanMX* selection. The genomic integration of TAP–*KanMX* was checked by PCR.

### Determinations of Plasmid Copy Numbers and TAP-tagged Proteins

The sample preparation scheme to determine plasmid copy numbers and protein levels is shown in Figure S1. Each TAP strain with either an empty vector or the corresponding TAP plasmid or Target plasmid was precultured in 6 mL of Edinburgh minimal medium (EMM) with leucine (for 24 h) or without leucine (for 48 h). Each sample (200 µL) was used to measure the plasmid copy number using real-time PCR as described [2]. An aliquot of 2.5 mL (+leucine) or 5.0 mL (−leucine) of the preculture medium was transferred to 10 mL of fresh EMM medium so that the optical density at 600 nm (OD_600_) was ≅0.5. After culture for 6 h, cells at 4 OD_600_ (i.e., if the OD600 was 1.0, cells were collected from 4 mL of culture) were collected. Cell proteins were extracted by treatment with 0.3 N NaOH for 10 min and then separated by SDS polyacrylamide gel electrophoresis (NuPage 4–12% Bis-Tris Gel, Invitrogen). We performed two electrophoretic separations for each sample. One gel was stained with Coomassie® G-250 (SimplyBlue™ SafeStain, Invtrogen), and the other gel was subjected to Western blotting using peroxidase-antiperoxidase (P1901l, Sigma-Aldrich) as described [1], [21]. The density of a 50 kDa band (corresponding to the size of tubulin) for each sample was measured from a scanned Coomassie-stained gel using the gel analysis option of ImageJ 1.44o software to estimate the total protein level for normalization. Intensity of the corresponding protein band on each Western blot was measured using an LAS-4000 image analyzer (Fujifilm/GE Healthcare); data within the linear detection range from among serially diluted samples were used (circled in Figure 3 and Figure S2). The fold-increase in protein level with an increase in the copy number was calculated as follows: *Dilution* × (*TAPint_TAP plasmid/TAPint_vector*)/(*Coom_TAP plasmid/Coom_vector*). *Dilution* indicates the fold-dilution of the sample. *TAPint_TAP plasmid* and *Coom_TAP plasmid* indicate the intensities of a detected TAP-tagged protein and that of the Coomassie-stained 50 kDa protein of the sample with a TAP plasmid. *TAPint_vector* and *Coom_vector* indicate the intensities of a sample with an empty vector.

### Quantification of mRNA

Total RNA of each strain was purified as described previously [22]. cDNA was synthesized from the RNA using PrimeScript® RT reagent Kit with gDNA Eraser (Takara) according to the manufacture’s protocol. The cDNA was subjected to the real-time PCR analysis using LightCycler® FastStart DNA Master SYBR Green I (Roche) for quantification of mRNA of each target gene. The PCR primers used were listed in Table S10. The relative increase in mRNA abundance for each target gene upon the TAP plasmid introduction (a TAP strain harboring a TAP plasmid) was calculated using the standard curve generated by a serial dilution of the control sample (a TAP strain harboring an empty vector). *nda3* gene was used to standardize the total mRNA levels.

## Supporting Information

Figure S1 **Sample preparation for determining plasmid copy numbers, mRNA (optional), and protein levels.** Figure details are provided in Methods in the main text.
(TIF)

Figure S2 **Results for measurements of protein fold-increases and copy numbers of cdc-TAP.** Circled numbers indicate the fold-dilutions used to measure Cdc-TAP protein intensities. Total proteins were visualized using Coomassie® G-250 staining. “Copy number*” indicates the plasmid copy number determined by real-time PCR plus 1 (genomic copy).
(TIF)

Table S1 **Fission yeast cell cycle regulatory genes analyzed in this study.**
(DOC)

Table S2 **Copy number limits for native- and TAP-tagged cdc genes.**
(DOC)

Table S3 **Fold increase in mRNA level upon copy number increase.**
(DOC)

Table S4 **Plasmids used in this study.**
(DOC)

Table S5 **Fission yeast strains used in this study.**
(DOC)

Table S6 **“Up-tag” primers for constructing TAP plasmids.**
(DOC)

Table S7 **“Down-tag” primers for constructing TAP plasmids.**
(DOC)

Table S8 **“Up-int” primers for constructing TAP strains.**
(DOC)

Table S9 **Other PCR primers.**
(DOC)

Table S10 **Primers used for mRNA quantification.**
(DOC)
