# Supplementary figures and images for: Relationships between Cell Cycle Regulator Gene Copy Numbers and Protein Expression Levels in Schizosaccharomyces pombe
Source: PLoS One. 2013 Sep 3;8(9):e73319. doi: 10.1371/journal.pone.0073319 (PMC3760898; doi:10.1371/journal.pone.0073319)

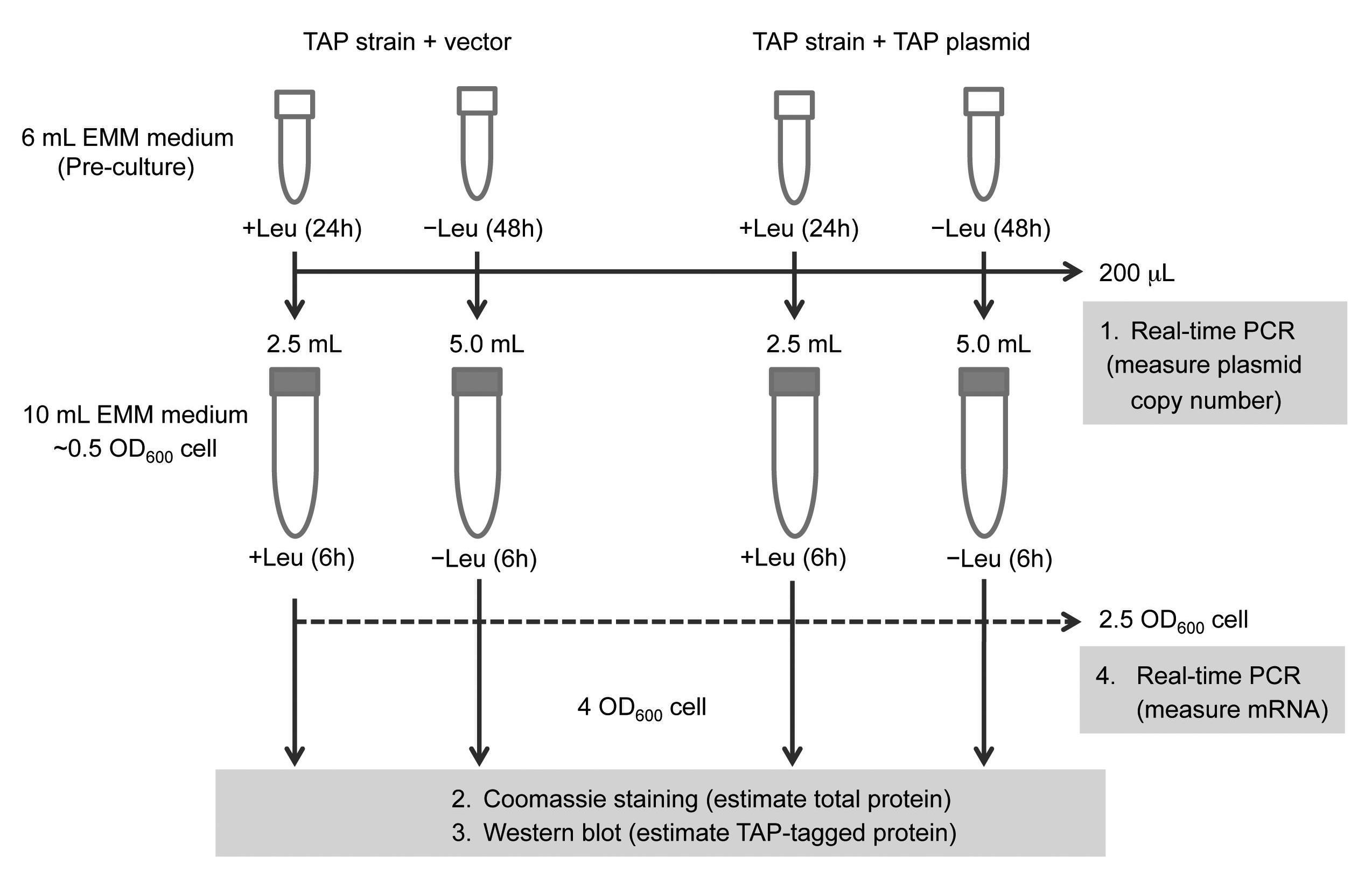

Supplement: Figure S1 — Sample preparation for determining plasmid copy numbers, mRNA (optional), and protein levels. Figure details are provided in Methods in the main text. (TIF) [file pone.0073319.s001.tif]

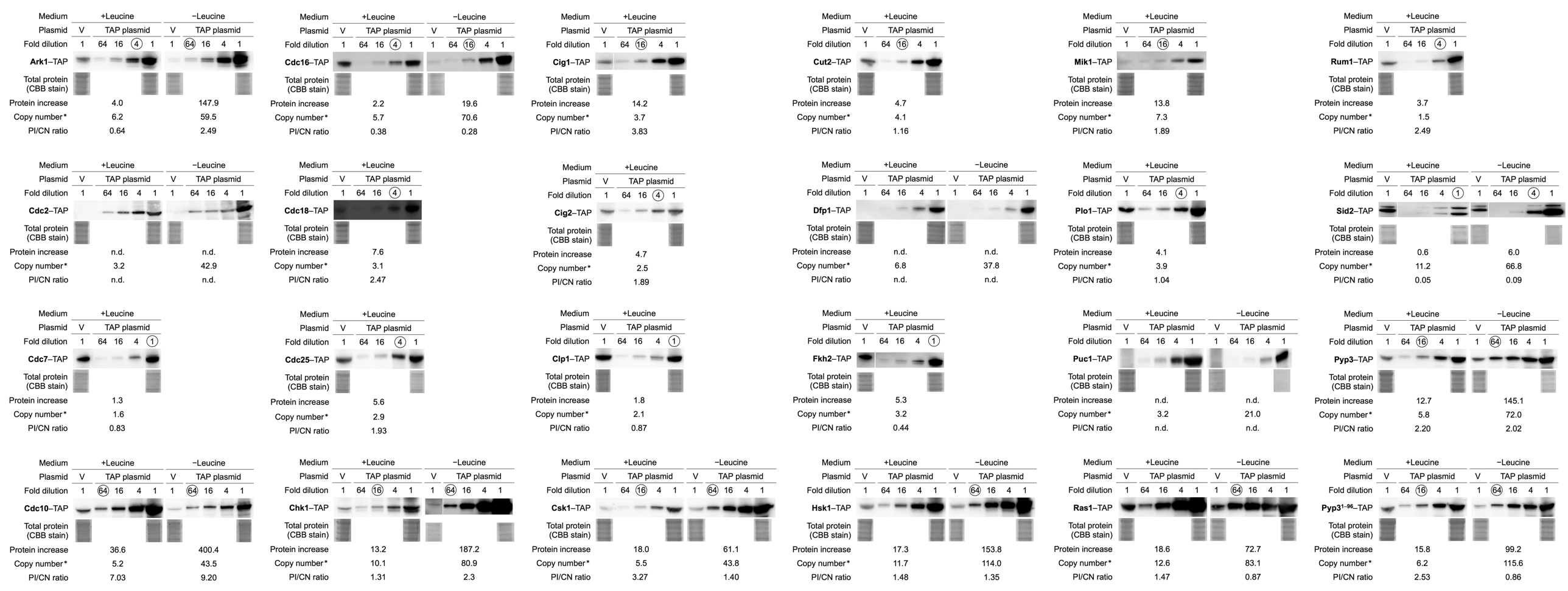

Supplement: Figure S2 — Results for measurements of protein fold-increases and copy numbers of cdc-TAP. Circled numbers indicate the fold-dilutions used to measure Cdc-TAP protein intensities. Total proteins were visualized using Coomassie® G-250 staining. “Copy number*” indicates the plasmid copy number determined by real-time PCR plus 1 (genomic copy). (TIF) [file pone.0073319.s002.tif]
