# Supplementary material for: Relationships between Cell Cycle Regulator Gene Copy Numbers and Protein Expression Levels in Schizosaccharomyces pombe
Source: PLoS One. 2013 Sep 3;8(9):e73319. doi: 10.1371/journal.pone.0073319 (PMC3760898; doi:10.1371/journal.pone.0073319)
Supplement: Table S1 — Fission yeast cell cycle regulatory genes analyzed in this study. (DOC) [file pone.0073319.s003.doc]

**Table S1**. Fission yeast cell cycle regulatory genes analyzed in this study

|  | Gene Name | Copy Number changed?^*1^ | TAP plasmid | | TAP strain | |
| --- | --- | --- | --- | --- | --- | --- |
|  |  |  | Construction succeeded?^*2^ | Protein detected?^*2^ | Construction succeeded?^*2^ | Protein detected?^*2^ |
| 1 | *ark1* |  |  |  |  |  |
| 2 | *cdc2* |  |  |  |  | No |
| 3 | *cdc7* |  |  |  |  |  |
| 4 | *cdc10* |  |  |  |  |  |
| 5 | *cdc13* |  |  | No | n.d. | n.d. |
| 6 | *cdc16* |  |  |  |  |  |
| 7 | *cdc18* |  |  |  |  |  |
| 8 | *cdc25* |  |  |  |  |  |
| 9 | *chk1* |  |  |  |  |  |
| 10 | *cig1* |  |  |  |  |  |
| 11 | *cig2* |  |  |  |  |  |
| 12 | *clp1* |  |  |  |  |  |
| 13 | *csk1* |  |  |  |  |  |
| 14 | *cut1* | Yes |  |  | No | n.d. |
| 15 | *cut2* |  |  |  |  |  |
| 16 | *dfp1* | Yes |  |  |  | No |
| 17 | *fkh2* |  |  |  |  |  |
| 18 | *hsk1* |  |  |  |  |  |
| 19 | *mik1* |  |  |  |  |  |
| 20 | *plo1* |  |  |  |  |  |
| 21 | *puc1* | Yes |  |  |  | No |
| 22 | *ras1* |  |  |  |  |  |
| 23 | *res1* |  |  | No | n.d. | n.d. |
| 24 | *res2* | Yes |  | No | n.d. | n.d. |
| 25 | *rum1* | Yes |  |  |  |  |
| 26 | *sid2* |  |  |  |  |  |
| 27 | *slp1* | Yes |  | No | n.d. | n.d. |
| 28 | *spg1* |  | No | n.d. | n.d. | n.d. |
| 29 | *srw1* |  |  | No | n.d. | n.d. |
| 30 | *wee1* |  | No | n.d. | n.d. | n.d. |
| 31 | *pyp3* |  |  |  |  |  |

n.d.: Construction or protein detection was not done.

*1. Blank indicates “No”

*2. Blank indicates “Yes”
