# Supplementary material for: Relationships between Cell Cycle Regulator Gene Copy Numbers and Protein Expression Levels in Schizosaccharomyces pombe
Source: PLoS One. 2013 Sep 3;8(9):e73319. doi: 10.1371/journal.pone.0073319 (PMC3760898; doi:10.1371/journal.pone.0073319)
Supplement: Table S2 — Copy number limits for native- and TAP-tagged cdc genes. (DOC) [file pone.0073319.s004.doc]

**Table S2**. Copy number limits for native- and TAP-tagged *cdc* genes

|  | Gene | TAP-tagged | | Native* | |
| --- | --- | --- | --- | --- | --- |
| 1 | *ark1* | 55.0 | ± 16.9 | 63.0 | ± 11.4 |
| 2 | *cdc2* | 38.8 | ± 8.3 | 52.1 | ± 8.8 |
| 3 | *cdc7* | 4.8 | ± 0.6 | 10.5 | ± 1.9 |
| 4 | *cdc10* | 67.6 | ± 11.5 | 66.1 | ± 7.9 |
| 5 | *cdc13* | 3.7 | ± 1.4 | 1.4 | ± 0.7 |
| 6 | *cdc16* | 44.1 | ± 8.5 | 48.2 | ± 3.5 |
| 7 | *cdc18* | 29.5 | ± 9.2 | 39.0 | ± 4.8 |
| 8 | *cdc25* | 31.9 | ± 12.6 | 38.7 | ± 15.1 |
| 9 | *chk1* | 74.0 | ± 15.7 | 55.9 | ± 4.6 |
| 10 | *cig1* | 39.6 | ± 15.4 | 42.8 | ± 20.1 |
| 11 | *cig2* | 17.4 | ± 6.2 | 14.9 | ± 4.7 |
| 12 | *clp1* | 3.9 | ± 4.1 | 1.6 | ± 1.0 |
| 13 | *csk1* | 82.2 | ± 8.2 | 77.7 | ± 9.6 |
| 14 | *cut1* | 7.1 | ± 12.9 | 56.6 | ± 21.3 |
| 15 | *cut2* | 19.3 | ± 10.3 | 32.2 | ± 4.6 |
| 16 | *dfp1* | 82.5 | ± 6.7 | 41.3 | ± 4.0 |
| 17 | *fkh2* | 6.7 | ± 0.3 | 24.5 | ± 2.2 |
| 18 | *hsk1* | 78.5 | ± 16.1 | 77.7 | ± 13.2 |
| 19 | *mik1* | 75.1 | ± 6.8 | 65.9 | ± 6.6 |
| 20 | *plo1* | 32.7 | ± 0.6 | 31.9 | ± 3.6 |
| 21 | *puc1* | 33.4 | ± 1.4 | 72.9 | ± 8.6 |
| 22 | *ras1* | 49.1 | ± 3.1 | 77.8 | ± 8.5 |
| 23 | *res1* | 30.7 | ± 1.6 | 28.3 | ± 7.4 |
| 24 | *res2* | 33.1 | ± 9.1 | 75.9 | ± 9.2 |
| 25 | *rum1* | 0.4 | ± 0.1 | 13.8 | ± 2.3 |
| 26 | *sid2* | 74.2 | ± 15.9 | 62.5 | ± 8.2 |
| 27 | *slp1* | 87.2 | ± 3.8 | 26.2 | ± 10.3 |
| 28 | *spg1* | ND | | 0.6 | ± 0.5 |
| 29 | *srw1* | 73.0 | ± 10.8 | 57.5 | ± 9.1 |
| 30 | *wee1* | ND | | 0.9 | ± 0.4 |
| 31 | *pyp3* | 76.7 | ± 15.1 | 60.5 | ± 7.2 |

Copy numbers under –leucine conditions are shown, except for *cdc13*, *clp1*, *spg1*, and *wee1* (copy numbers under +leucine conditions are shown for these genes). * Copy number data for native genes were from [1].
