# Supplementary material for: Relationships between Cell Cycle Regulator Gene Copy Numbers and Protein Expression Levels in Schizosaccharomyces pombe
Source: PLoS One. 2013 Sep 3;8(9):e73319. doi: 10.1371/journal.pone.0073319 (PMC3760898; doi:10.1371/journal.pone.0073319)
Supplement: Table S3 — Fold increase in mRNA level upon copy number increase. (DOC) [file pone.0073319.s005.doc]

**Table S3. Fold increase in mRNA level upon copy number increase**

| Gene | +Leucine | −Leucine |
| --- | --- | --- |
| *cdc16* | 7.3 ± 0.8 | 93.3 ± 4.9 |
| *sid2* | 21.8 ± 3.1 | 112.3 ± 5.1 |
| *cdc10* | 14.8 ± 4.0 | 321.7 ± 22.2 |
| *cig1* | 12.5 ± 0.7 |  |
