# Supplementary material for: Relationships between Cell Cycle Regulator Gene Copy Numbers and Protein Expression Levels in Schizosaccharomyces pombe
Source: PLoS One. 2013 Sep 3;8(9):e73319. doi: 10.1371/journal.pone.0073319 (PMC3760898; doi:10.1371/journal.pone.0073319)
Supplement: Table S4 — Plasmids used in this study. (DOC) [file pone.0073319.s006.doc]

## **Table S4**. Plasmids used in this study

| Name | Description | Source |
| --- | --- | --- |
| pTOWspdh5-M | *ColE1ori*, *AmpR*, *ars3002×2*, *ura4-EGFP*, *leu2-89* | [3] |
| pTOW-ark1T | pTOWspdh5-M containing *ark1–TAP–KanMX4* | This study |
| pTOW-cdc7T | pTOWspdh5-M containing *cdc7–TAP–KanMX4* | This study |
| pTOW-cdc10T | pTOWspdh5-M containing *cdc10–TAP–KanMX4* | This study |
| pTOW-cdc13T | pTOWspdh5-M containing *cdc13–TAP–KanMX4* | This study |
| pTOW-cdc16T | pTOWspdh5-M containing *cdc16–TAP–KanMX4* | This study |
| pTOW-cdc18T | pTOWspdh5-M containing *cdc18–TAP–KanMX4* | This study |
| pTOW-cdc25T | pTOWspdh5-M containing *cdc25–TAP–KanMX4* | This study |
| pTOW-chk1T | pTOWspdh5-M containing *chk1–TAP–KanMX4* | This study |
| pTOW-cig1T | pTOWspdh5-M containing *cig1–TAP–KanMX4* | This study |
| pTOW-cig2T | pTOWspdh5-M containing *cig2–TAP–KanMX4* | This study |
| pTOW-clp1T | pTOWspdh5-M containing *clp1–TAP–KanMX4* | This study |
| pTOW-csk1T | pTOWspdh5-M containing *csk1–TAP–KanMX4* | This study |
| pTOW-cug2T | pTOWspdh5-M containing *cut2–TAP–KanMX4* | This study |
| pTOW-fkh2T | pTOWspdh5-M containing *fkh2–TAP–KanMX4* | This study |
| pTOW-hsk1T | pTOWspdh5-M containing *hsk1–TAP–KanMX4* | This study |
| pTOW-mik1T | pTOWspdh5-M containing *mik1–TAP–KanMX4* | This study |
| pTOW-plo1T | pTOWspdh5-M containing *plo1–TAP–KanMX*4 | This study |
| pTOW-ras1T | pTOWspdh5-M containing *ras1–TAP–KanMX4* | This study |
| pTOW-rum1T | pTOWspdh5-M containing *rum1–TAP–KanMX4* | This study |
| pTOW-sid2T | pTOWspdh5-M containing *sid2–TAP–KanMX4* | This study |
| pTOW-pyp3T | pTOWspdh5-M containing *pyp3–TAP–KanMX4* | This study |
| pTOW-pyp3^1–96^T | pTOWspdh5-M containing *pyp31–96–TAP–KanMX4* | This study |
| pTOWsp-M-*cdc10* | pTOWsp-M containing *cdc10* | [2] |
| pTOWsp-M-*cdc16* | pTOWsp-M containing *cdc16* | [2] |
| pTOWsp-M-*cig1* | pTOWsp-M containing *cig1* | [2] |
| pTOWsp-M-*sid2* | pTOWsp-M containing *sid2* | [2] |
