# Supplementary material for: Relationships between Cell Cycle Regulator Gene Copy Numbers and Protein Expression Levels in Schizosaccharomyces pombe
Source: PLoS One. 2013 Sep 3;8(9):e73319. doi: 10.1371/journal.pone.0073319 (PMC3760898; doi:10.1371/journal.pone.0073319)
Supplement: Table S5 — Fission yeast strains used in this study. (DOC) [file pone.0073319.s007.doc]

## **Table S5**. Fission yeast strains used in this study

| Name | Genotype | Source |
| --- | --- | --- |
| FY7652 | *h- leu1-32 ura4-D18* | NBRPyeast |
| AC0001 | *h- leu1-32 ura4-D18 ark1::ark1–TAP–KanMX4* | This study |
| AC0002 | *h- leu1-32 ura4-D18 cdc7::cdc7–TAP–KanMX4* | This study |
| AC0003 | *h- leu1-32 ura4-D18 cdc10::cdc10–TAP–KanMX4* | This study |
| AC0004 | *h- leu1-32 ura4-D18 cdc13::cdc13–TAP–KanMX4* | This study |
| AC0005 | *h- leu1-32 ura4-D18 cdc16::cdc16–TAP–KanMX4* | This study |
| AC0006 | *h- leu1-32 ura4-D18 cdc18::cdc18–TAP–KanMX4* | This study |
| AC0007 | *h- leu1-32 ura4-D18 cdc25::cdc25–TAP–KanMX4* | This study |
| AC0008 | *h- leu1-32 ura4-D18 chk1::chk1–TAP–KanMX4* | This study |
| AC0009 | *h- leu1-32 ura4-D18 cig1::cig1–TAP–KanMX4* | This study |
| AC0010 | *h- leu1-32 ura4-D18 cig2::cig2–TAP–KanMX4* | This study |
| AC0011 | *h- leu1-32 ura4-D18 clp1::clp1–TAP–KanMX4* | This study |
| AC0012 | *h- leu1-32 ura4-D18 csk1::csk1–TAP–KanMX4* | This study |
| AC0013 | *h- leu1-32 ura4-D18 cut2::cut2–TAP–KanMX4* | This study |
| AC0014 | *h- leu1-32 ura4-D18 fkh2::fkh2–TAP–KanMX4* | This study |
| AC0015 | *h- leu1-32 ura4-D18 hsk1::hsk1–TAP–KanMX4* | This study |
| AC0016 | *h- leu1-32 ura4-D18 mik1::mik1–TAP–KanMX4* | This study |
| AC0017 | *h- leu1-32 ura4-D18 plo1::plo1–TAP–KanMX4* | This study |
| AC0018 | *h- leu1-32 ura4-D18 ras1::ras1–TAP–KanMX4* | This study |
| AC0019 | *h- leu1-32 ura4-D18 rum1::rum1–TAP–KanMX4* | This study |
| AC0020 | *h- leu1-32 ura4-D18 sid2::sid2–TAP–KanMX4* | This study |
| AC0021 | *h- leu1-32 ura4-D18 pyp3::pyp3–TAP–KanMX4* | This study |
| AC0023 | *h- leu1-32 ura4-D18 pyp3::pyp3^1–96^–TAP–KanMX4* | This study |
