# Supplementary material for: Relationships between Cell Cycle Regulator Gene Copy Numbers and Protein Expression Levels in Schizosaccharomyces pombe
Source: PLoS One. 2013 Sep 3;8(9):e73319. doi: 10.1371/journal.pone.0073319 (PMC3760898; doi:10.1371/journal.pone.0073319)
Supplement: Table S6 — “Up-tag” primers for constructing TAP plasmids. (DOC) [file pone.0073319.s008.doc]

**Table S6**. “Up-tag” primers for constructing TAP plasmids

|  | Gene | Name | Sequence (5′ to 3′) |
| --- | --- | --- | --- |
| 1 | *ark1* | OHM27 | TATCCTCCTCGCCCTTGCTCACCATATGGGAAGATTCAGAACTTTTGC |
| 2 | *cdc2* | OHM29 | TATCCTCCTCGCCCTTGCTCACCATATGATGAAAATCACGAAGATAAT |
| 3 | *cdc7* | OHM31 | TATCCTCCTCGCCCTTGCTCACCATATGCTGCGTTAATGGCTGCTTTG |
| 4 | *cdc10* | OHM33 | TATCCTCCTCGCCCTTGCTCACCATATGTGCTTGATGTTCTTTAACAA |
| 5 | *cdc13* | OHM35 | TATCCTCCTCGCCCTTGCTCACCATATGCCATTCTTCATCTTTCATGT |
| 6 | *cdc16* | OHM37 | TATCCTCCTCGCCCTTGCTCACCATATGTGTTAGTCGGTCAATCAGAA |
| 7 | *cdc18* | OHM39 | TATCCTCCTCGCCCTTGCTCACCATATGTCTTCTGTCAAAAAATCGTT |
| 8 | *cdc25* | OHM41 | TATCCTCCTCGCCCTTGCTCACCATATGAAATCTTCTAAGTGTAGAGA |
| 9 | *chk1* | OHM43 | TATCCTCCTCGCCCTTGCTCACCATATGATTTTGTGAAACATCTGTAA |
| 10 | *cig1* | OHM45 | TATCCTCCTCGCCCTTGCTCACCATATGAATCACACTTAGTACCCAGT |
| 11 | *cig2* | OHM47 | TATCCTCCTCGCCCTTGCTCACCATATGGTGACCATCATTTGTTAAAG |
| 12 | *clp1* | OHM49 | TATCCTCCTCGCCCTTGCTCACCATATGAGAAATTAGCCGGCTTTTAG |
| 13 | *csk1* | OHM51 | TATCCTCCTCGCCCTTGCTCACCATATGTGCATATTGTGAAAGCCTAG |
| 14 | *cut1* | OHM53 | TATCCTCCTCGCCCTTGCTCACCATATGTGGAATAATATAAGCAGGTAT |
| 15 | *cut2* | OHM55 | TATCCTCCTCGCCCTTGCTCACCATATGTAACAATCCTGTATCCAAAG |
| 16 | *dfp1* | OHM57 | TATCCTCCTCGCCCTTGCTCACCATATGATCTGGCCTTAAGGGACGTT |
| 17 | *fkh2* | OHM59 | TATCCTCCTCGCCCTTGCTCACCATATGAGCACTACTTTTAACATTAGA |
| 18 | *hsk1* | OHM61 | TATCCTCCTCGCCCTTGCTCACCATATGAGCTCCATCCTGCAAAGCATC |
| 19 | *mik1* | OHM63 | TATCCTCCTCGCCCTTGCTCACCATATGAGTTTCTAACCAACTGTTATG |
| 20 | *plo1* | OHM65 | TATCCTCCTCGCCCTTGCTCACCATATGACTCACTTCCATTTTCGACG |
| 21 | *puc1* | OHM67 | TATCCTCCTCGCCCTTGCTCACCATATGCAAAGTACGCTCAGTATCCT |
| 22 | *ras1* | OHM69 | TATCCTCCTCGCCCTTGCTCACCATATGACATATAACACAACATTTAG |
| 23 | *res1* | OHM71 | TATCCTCCTCGCCCTTGCTCACCATATGAGATCCACTTTGATCTGTAT |
| 24 | *res2* | OHM73 | TATCCTCCTCGCCCTTGCTCACCATATGTTTTTCTCGGGTTAATG |
| 25 | *rum1* | OHM75 | TATCCTCCTCGCCCTTGCTCACCATATGTCGTAATAAATTGTGCCTGT |
| 26 | *sid2* | OHM77 | TATCCTCCTCGCCCTTGCTCACCATATGTAATAGAGTCCCGAAAGAAGGAG |
| 27 | *slp1* | OHM79 | TATCCTCCTCGCCCTTGCTCACCATATGACGGATTGTTATGCTGCTGG |
| 28 | *spg1* | OHM81 | TATCCTCCTCGCCCTTGCTCACCATATGGCGATCGATGTATTCCAAAAT |
| 29 | *srw1* | OHM83 | TATCCTCCTCGCCCTTGCTCACCATATGCCGTATTTTCATTGTAGGGT |
| 30 | *wee1* | OHM85 | TATCCTCCTCGCCCTTGCTCACCATATGAACATTCACCTGCCAATCTT |
| 31 | *pyp3* | OHM87 | TATCCTCCTCGCCCTTGCTCACCATATGTAACTGAGGAAGAAGAAATTC |
| 32 | *pyp^31–96^* | OHM87-1 | TATCCTCCTCGCCCTTGCTCACCATATGTGTTACAAAAAATGATGAAA |
