# Supplementary material for: Relationships between Cell Cycle Regulator Gene Copy Numbers and Protein Expression Levels in Schizosaccharomyces pombe
Source: PLoS One. 2013 Sep 3;8(9):e73319. doi: 10.1371/journal.pone.0073319 (PMC3760898; doi:10.1371/journal.pone.0073319)
Supplement: Table S7 — “Down-tag” primers for constructing TAP plasmids. (DOC) [file pone.0073319.s009.doc]

**Table S7**. “Down-tag” primers for constructing TAP plasmids

|  | Gene | Name | Sequence (5′ to 3′) |
| --- | --- | --- | --- |
| 1 | *ark1* | OHM28 | CCGGCGGCATGGACGAGCTGTACAAGCTTTAACCGCCATCTTGGTACTT |
| 2 | *cdc2* | OHM30 | CCGGCGGCATGGACGAGCTGTACAAGCTTTAATTTTCGTCTCTTATTAT |
| 3 | *cdc7* | OHM32 | CCGGCGGCATGGACGAGCTGTACAAGCTTTGAACACTATTAAACGCATT |
| 4 | *cdc10* | OHM34 | CCGGCGGCATGGACGAGCTGTACAAGCTTTAATATTGCTTTTTGTGGTT |
| 5 | *cdc13* | OHM36 | CCGGCGGCATGGACGAGCTGTACAAGCTTTAATTTAGTGTATTGTGCAT |
| 6 | *cdc16* | OHM38 | CCGGCGGCATGGACGAGCTGTACAAGCTTTAATACTAGGGTAGGGTTTT |
| 7 | *cdc18* | OHM40 | CCGGCGGCATGGACGAGCTGTACAAGCTTTAGTACTATCATTTCTTTCT |
| 8 | *cdc25* | OHM42 | CCGGCGGCATGGACGAGCTGTACAAGCTTTAATGATTTTAGGCTGACTC |
| 9 | *chk1* | OHM44 | CCGGCGGCATGGACGAGCTGTACAAGCTTTAATTGCACATCTTTTGAAA |
| 10 | *cig1* | OHM46 | CCGGCGGCATGGACGAGCTGTACAAGCTTTGAGTTTGCTTTCAGAAGTT |
| 11 | *cig2* | OHM48 | CCGGCGGCATGGACGAGCTGTACAAGCTTTAACGAACGCTCTTATAAAT |
| 12 | *clp1* | OHM50 | CCGGCGGCATGGACGAGCTGTACAAGCTTTAATAAACCTGTAATTACTG |
| 13 | *csk1* | OHM52 | CCGGCGGCATGGACGAGCTGTACAAGCTTTAAAATTTACTCTCAGGATT |
| 14 | *cut1* | OHM54 | CCGGCGGCATGGACGAGCTGTACAAGCTTTAAACTGTCTAAAATTCTTA |
| 15 | *cut2* | OHM56 | CCGGCGGCATGGACGAGCTGTACAAGCTTTAAAAAGATTCCGAATTTTC |
| 16 | *dfp1* | OHM58 | CCGGCGGCATGGACGAGCTGTACAAGCTTTGAGAAAATAGCCCGTGTCT |
| 17 | *fkh2* | OHM60 | CCGGCGGCATGGACGAGCTGTACAAGCTTTAATGCCAACAAATTCACCT |
| 18 | *hsk1* | OHM62 | CCGGCGGCATGGACGAGCTGTACAAGCTTTGAGAAGATTTGCTGGCAAT |
| 19 | *mik1* | OHM64 | CCGGCGGCATGGACGAGCTGTACAAGCTTTGAGGGATTCTGTGTGCGAA |
| 20 | *plo1* | OHM66 | CCGGCGGCATGGACGAGCTGTACAAGCTTTAATAATACCGTTAATCTAT |
| 21 | *puc1* | OHM68 | CCGGCGGCATGGACGAGCTGTACAAGCTTTAACTTTAACATTGCTTCTT |
| 22 | *ras1* | OHM70 | CCGGCGGCATGGACGAGCTGTACAAGCTTTAGCAAGTATTATTGCAGAA |
| 23 | *res1* | OHM72 | CCGGCGGCATGGACGAGCTGTACAAGCTTTAATTTTTTTGGTTTTAAAT |
| 24 | *res2* | OHM74 | CCGGCGGCATGGACGAGCTGTACAAGCTTTGAATCTTGGAAACTTTCATTTA |
| 25 | *rum1* | OHM76 | CCGGCGGCATGGACGAGCTGTACAAGCTTTAACTTTTTTTCGCATTTTG |
| 26 | *sid2* | OHM78 | CCGGCGGCATGGACGAGCTGTACAAGCTTTAATCAAAGGGAAATTT |
| 27 | *slp1* | OHM80 | CCGGCGGCATGGACGAGCTGTACAAGCTTTGAACAACACCAGTTTCTTT |
| 28 | *spg1* | OHM82 | CCGGCGGCATGGACGAGCTGTACAAGCTTTGATTTTTAATGCTTTACCA |
| 29 | *srw1* | OHM84 | CCGGCGGCATGGACGAGCTGTACAAGCTTTAATGCAACACATTCACTCT |
| 30 | *wee1* | OHM86 | CCGGCGGCATGGACGAGCTGTACAAGCTTTAAACCTTTTAGAGACTCTT |
| 31 | *pyp3* | OHM88 | CCGGCGGCATGGACGAGCTGTACAAGCTTTAGCCTTTGGTTTAGAGTTT |
| 32 | *pyp^31–96^* | OHM88-1 | CCGGCGGCATGGACGAGCTGTACAAGCTTTAATTACTAACTTAGTCTTC |
