# Supplementary material for: Relationships between Cell Cycle Regulator Gene Copy Numbers and Protein Expression Levels in Schizosaccharomyces pombe
Source: PLoS One. 2013 Sep 3;8(9):e73319. doi: 10.1371/journal.pone.0073319 (PMC3760898; doi:10.1371/journal.pone.0073319)
Supplement: Table S8 — “Up-int” primers for constructing TAP strains. (DOC) [file pone.0073319.s010.doc]

**Table S8**. “Up-int” primers for constructing TAP strains

|  | Gene | Name | Sequence (5′ to 3′) |
| --- | --- | --- | --- |
| 1 | *ark1* | OHM0183 | GGTGGAGGGAAAAGAGCATA |
| 2 | *cdc2* | OHM0184 | CCCGGTGACTCTGAGATCGAC |
| 3 | *cdc7* | OHM0185 | AAAGAAAAGTTAACTCATAA |
| 4 | *cdc10* | OHM0186 | ACTGTCAATGAAAACAACAA |
| 5 | *cdc13* | OHM0187 | TACCTTGCCAGGGAAATGCT |
| 6 | *cdc16* | OHM0188 | CCGAGAGCAGCTCATCGATC |
| 7 | *cdc18* | OHM0189 | CTTCAACAGAAAGCCATCCT |
| 8 | *cdc25* | OHM0190 | GACAGACGAATGAATAGTCA |
| 9 | *chk1* | OHM0191 | GACAGTCTACGACTACTTGC |
| 10 | *cig1* | OHM0192 | GCCAAATATCTTCAAGAAGT |
| 11 | *cig2* | OHM0193 | GCTGCCGCAATGTATTTGAG |
| 12 | *clp1* | OHM0194 | GATTCAGAAATACAAAATGA |
| 13 | *csk1* | OHM0195 | GCAGGATCCGTTTACCTTAT |
| 14 | *cut1* | OHM0196 | ACTACATTCAATCAACTGGA |
| 15 | *cut2* | OHM0197 | ACAACACCCGCTACCTTGAA |
| 16 | *dfp1* | OHM0198 | AGAGATATCGCAGAGTTGAA |
| 17 | *fkh2* | OHM0199 | AAGCAAGCCAAGGAAATGGA |
| 18 | *hsk1* | OHM0200 | GACTGTAACAAAAGGATTTC |
| 19 | *mik1* | OHM0201 | GAAAATGGTGTTGAATGGCA |
| 20 | *plo1* | OHM0202 | ACTTCCAATACCATGCTTTTCATG |
| 21 | *puc1* | OHM0203 | AACATCGTAAACGAACATGT |
| 22 | *ras1* | OHM0204 | CGTGTAGTTTCAAGAGCTGA |
| 23 | *res1* | OHM0205 | GTAACTTACTTTTCTCAAATATGGAG |
| 24 | *res2* | OHM0206 | GACGGACTTTCGTTAAATAACGA |
| 25 | *rum1* | OHM0207 | AAGCCCAAACTCTTGTTTGC |
| 26 | *sid2* | OHM0208 | CGTACAGCATATCGTCCTCC |
| 27 | *slp1* | OHM0209 | CACTTCACTGATTTGGAGCCC |
| 28 | *spg1* | OHM0210 | ATCAAGAAGAGATTACCAAACAG |
| 29 | *srw1* | OHM0211 | TTGTTGTGGTCAAAGCAAAC |
| 30 | *wee1* | OHM0212 | TCTTCAACAGACAACGGTTC |
| 31 | *pyp3* | OHM0213 | TCATATTCTCCGGACTTTGA |
| 32 | *pyp^31–96^* | OSBI0717 | AGCTATGACCATGATTACGCCAAGCTTGTAAAAGG  GTGGATGGATATATA |
