# Supplementary material for: Relationships between Cell Cycle Regulator Gene Copy Numbers and Protein Expression Levels in Schizosaccharomyces pombe
Source: PLoS One. 2013 Sep 3;8(9):e73319. doi: 10.1371/journal.pone.0073319 (PMC3760898; doi:10.1371/journal.pone.0073319)
Supplement: Table S9 — Other PCR primers. (DOC) [file pone.0073319.s011.doc]

## **Table S9**. Other PCR primers

| Name | Sequence (5′ to 3′) |
| --- | --- |
| OHM179 | ATGGTGAGCAAGGGCGAGGAGGATATGGAAAAGAGAAGATGGAAA |
| OHM182 | CTTGTACAGCTCGTCCATGCCGCCGGTCGATGAATTCGAGCTCGT |
| OHM844 | AAGTTCTTGAAAACAAGAATCTTTTTATTGTCAGTACTCTTTATTTGTACAATTCATCCATACCATGGGTAATACCAGCA |
| OHM845 | TGCTGGTATTACCCATGGTATGGATGAATTGTACAAATAAAGAGTACTGACAATAAAAAGATTCTTGTTTTCAAGAACTT |
| OSBI870 | GGTGGAGGGAAAAGAGCATA |
