# Supplementary material for: Relationships between Cell Cycle Regulator Gene Copy Numbers and Protein Expression Levels in Schizosaccharomyces pombe
Source: PLoS One. 2013 Sep 3;8(9):e73319. doi: 10.1371/journal.pone.0073319 (PMC3760898; doi:10.1371/journal.pone.0073319)
Supplement: Table S10 — Primers used for mRNA quantification. (DOC) [file pone.0073319.s012.doc]

## **Table S10. Primers used for mRNA quantification**

| Target gene | Position | Sequence (5′ to 3′) |
| --- | --- | --- |
| *nda3* | Up | GCTGATGAGCATGGTTTG |
|  | Down | GACTTCACGGCATCCATAG |
| *cdc10* | Up | CTCTCCACCTCTCCACCTAT |
|  | Down | GTTCAACCTTGGCGTATCTG |
| *cdc16* | Up | ATGAATGCCCTCTATATGTCCT |
|  | Down | GGCAGTACAAGCACTCAAAGTAAG |
| *cig1* | Up | TCTTCACCGTTGACGAAATC |
|  | Down | ATGCGAATGTCGTGATCGTA |
| *sid2* | Up | GCGTGATATCCTTACGACAG |
|  | Down | AGTACGGAAATCACCACCAG |
